# Reliability of pain intensity clamping using response-dependent thermal stimulation in healthy volunteers

**DOI:** 10.1186/s12868-015-0164-4

**Published:** 2015-04-18

**Authors:** Yenisel Cruz-Almeida, Kelly M Naugle, Charles J Vierck, Roger B Fillingim, Joseph L Riley

**Affiliations:** Institute on Aging, University of Florida, Gainesville, FL USA; Cognitive Aging & Memory, University of Florida, Gainesville, FL USA; Pain Research and Intervention Center of Excellence, Gainesville, USA; Department of Aging & Geriatric Research, College of Medicine, University of Florida, Gainesville, FL USA; Department of Neuroscience, College of Medicine, University of Florida, Gainesville, FL USA; Department of Community Dentistry & Behavioral Sciences, College of Dentistry, University of Florida, Gainesville, FL USA; Department of Kinesiology, IUPUI, Indianapolis, IN USA

**Keywords:** Quantitative sensory testing, Pain sensitivity, Reliability

## Abstract

**Background:**

Pain intensity clamping uses the *RE*sponse-*D*ependent *S*timulation (REDSTIM) methodology to automatically adjust stimulus intensity to maintain a desired pain rating set-point which is continuously monitored from a subject’s real-time pain ratings. REDSTIM blinds subjects regarding the pain intensity set-point, supporting its use for assessing intervention efficacy. By maintaining the pain intensity at a constant level, a potential decrease in pain sensitivity can be detected by an increase in thermode temperature (unknown to the subject) and not by pain ratings alone. Further, previously described sensitizing and desensitizing trends within REDSTIM provide a novel insight into human pain mechanisms overcoming limitations of conventional testing methods. The purpose of the present study was to assess the test-retest reliability of pain intensity clamping using REDSTIM during three separate sessions.

**Methods:**

We used a method for testing changes in pain sensitivity of human subjects (REDSTIM) where the stimulus temperature is modulated to clamp pain intensity near a desired set-point. Temperature serves as the response variable and is used to infer pain sensitivity. Several measures were analyzed for reliability including average temperature and area under the curve (AUC). Intraclass correlation coefficients were calculated for each measure at pain rating set-points of 20/100 and 35/100.

**Results:**

Sixteen healthy individuals (mean age = 21.6 ± 3.9) participated in three experiments two days apart at both pain rating set-points. Most reliability coefficients were in the moderate to substantial range (r’s = 0.79 to 0.94) except for the negative AUC (r = 0.52), but only at the 20/100 pain rating set-point.

**Conclusions:**

The present study supports the test-retest reliability of pain intensity clamping using the REDSTIM methodology while providing a novel tool to examine human pain modulatory mechanisms and overcoming common shortcomings of conventional quantitative sensory testing methods.

## Background

To date, the majority of experimental pain studies using quantitative sensory testing (QST) methodology have relied on very short duration stimuli, which do not vary in intensity, but can be adjusted to different intensities per trial (i.e., thermal pain, mechanical pressure). Other QST procedures that allow for prolonged exposures may have changes in stimulus intensity that are out of the control of the experimenter (i.e., cold pressor task). Pain intensity clamping using the *RE*sponse-*D*ependent *S*timulation (REDSTIM) methodology was recently introduced as a new psychophysical approach to assess pain sensitivity in human subjects using prolonged thermal stimulation [1,2]. REDSTIM automatically adjusts stimulus intensity to maintain a desired pain rating set-point by continuously monitoring the subject’s real-time pain ratings. REDSTIM allows the maintenance of sustained but tolerable pain levels by varying stimulus intensity, which permits long trials of continuous stimulation even in the most pain-sensitive subjects. REDSTIM allows researchers to challenge the pain system with longer stimulation exposure times, testing slowly responding pain modulatory mechanisms. All REDSTIM stimuli are administered with subjects blinded to stimulus parameters while avoiding direct subject-experimenter interactions. REDSTIM also overcomes the drawback of administering a predetermined stimulus magnitude when the subject populations include individuals with vastly different pain sensitivities. The REDSTIM paradigm significantly overcomes important confounds in human pain experimentation, which are currently unavoidable with conventional QST methods [3].

During prolonged sequences of REDSTIM, pain intensity ratings oscillate around the desired set-point with similar temperature oscillations [1,2]. Previous work revealed that REDSTIM oscillations during ascending and descending series of stimuli yielded highly significant bidirectional sensitization and desensitization trends in healthy individuals [1]. Once the pain ratings exceed the set-point, the stimulator algorithm starts a descending stimulus series (i.e., temperature decrease) which reveals a *sensitizing trend* (i.e., continued increase in pain ratings despite decreases in temperature). Conversely, once the pain ratings go below the set-point, the algorithm starts an increasing stimulus series (i.e., temperature increase) revealing a *desensitizing trend* (i.e., continued decrease in pain ratings despite increases in temperature). In previous work we have used the sensitizing/desensitizing trend terminology because the pain ratings are *trending* in the opposite direction of the stimulus temperature. This is not to be confused with the definitions of sensitization and desensitization, as we have also shown that the average running temperature does not change across REDSTIM trials, suggesting that individuals do not experience prolonged sensitization or desensitization [4]. Although the exact mechanisms underlying these trend effects are not clear, previous work has shown that a desensitizing trend effect can be established robustly by a one-step decrease in thermal stimulation, termed offset analgesia [5]. Additionally, expectation-mediated mechanisms, such as those involved in placebo and nocebo effects, may also contribute. Thus, the patterns of these sensitizing and desensitizing trends likely reflect modulation of somatosensory input, which may provide novel insights into human pain mechanisms.

An important feature of an outcome measure, for both research and clinical purposes, is its reliable reproduction under the same conditions. A recent review points to the considerable variability in reliability estimates of currently used thermal QST measures with reliability coefficients ranging from fair to excellent [3]. To date, there are a limited number of studies using REDSTIM methodology in healthy [1,2,4] and diseased human subjects [6], with no studies examining its psychometric properties including reliability. Only when appropriate psychometric studies including reliability are conducted on a measure, can it be utilized as a behavioral marker to quantify therapeutic response to treatment [7]. REDSTIM could be specifically useful in drug trials where subject blinding to changes in pain sensitivity are critical for the study’s internal validity. Therefore, the present investigation was designed to examine the test-retest reliability of the REDSTIM measures across three different days at two different pain rating set-points.

## Results

**Figure 1:**
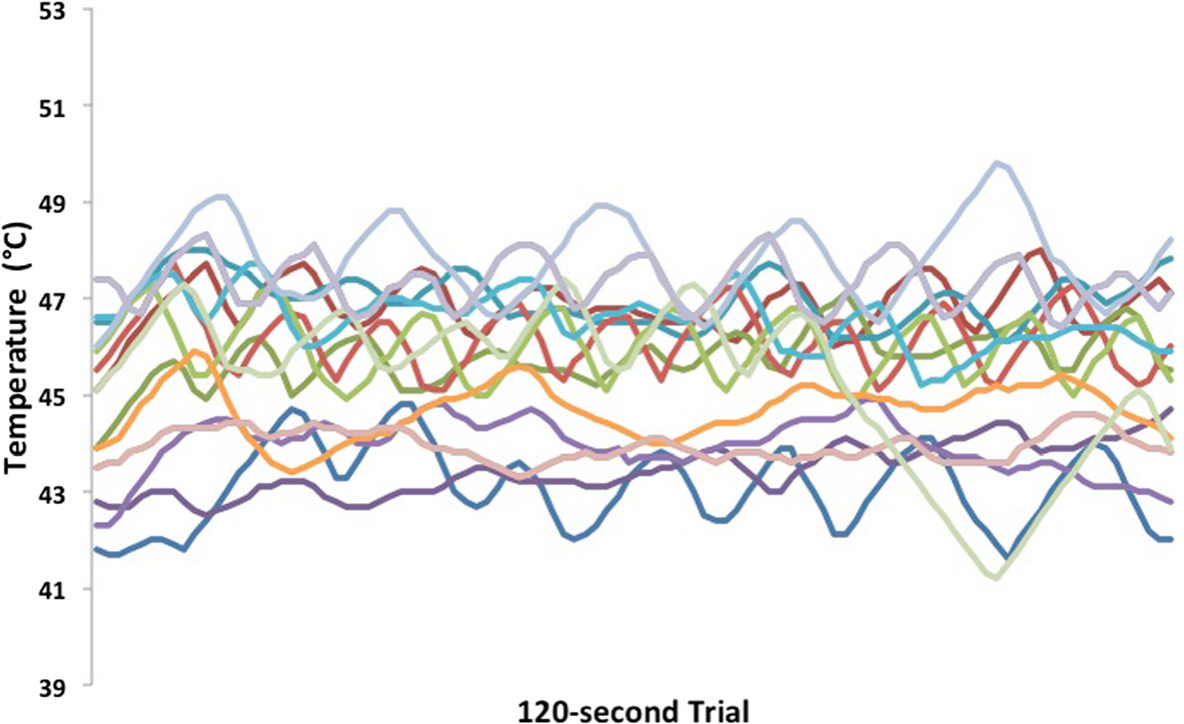
Measures obtained from the REDSTIM cycles.

**Figure 2:**
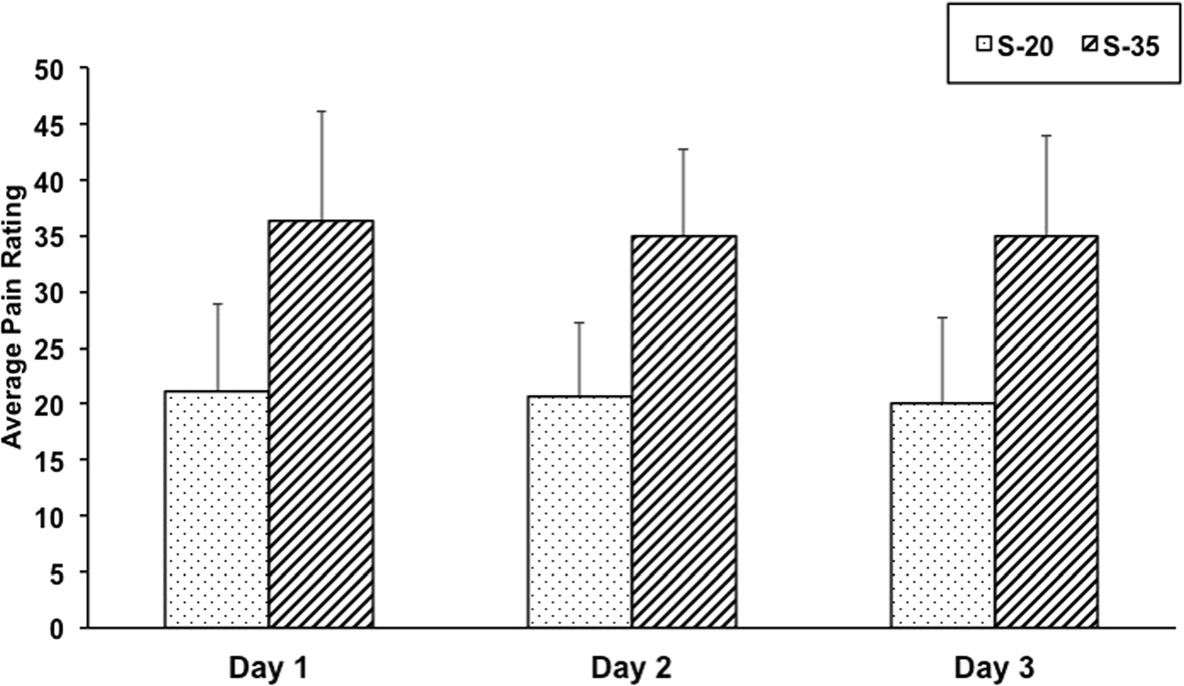
Average pain ratings across the three days at set-points of 20/100 (S-20) and 35/100 (s-35).

Sixteen healthy individuals (9 females) ranging in age from 20 to 35 years of age (mean = 21.6, SD = 3.9) participated in three different sessions two days apart. As previously reported [1], all subjects experienced temperature oscillations during REDSTIM stimulation on all three days (Figure 1 shows all subjects on day 2 of testing at the 35/100 pain rating set-point). Across the three days, the average pain rating was not significantly different from the preset pain ratings of 20 and 35 out 100 (Figure 2). To assess the reliability of the REDSTIM measures, several variables were extracted from the output during the 120-second maintenance phase of stimulation. Results are presented by the individual variables obtained from the REDSTIM output.

### Test-retest reliability

**Figure 3:**
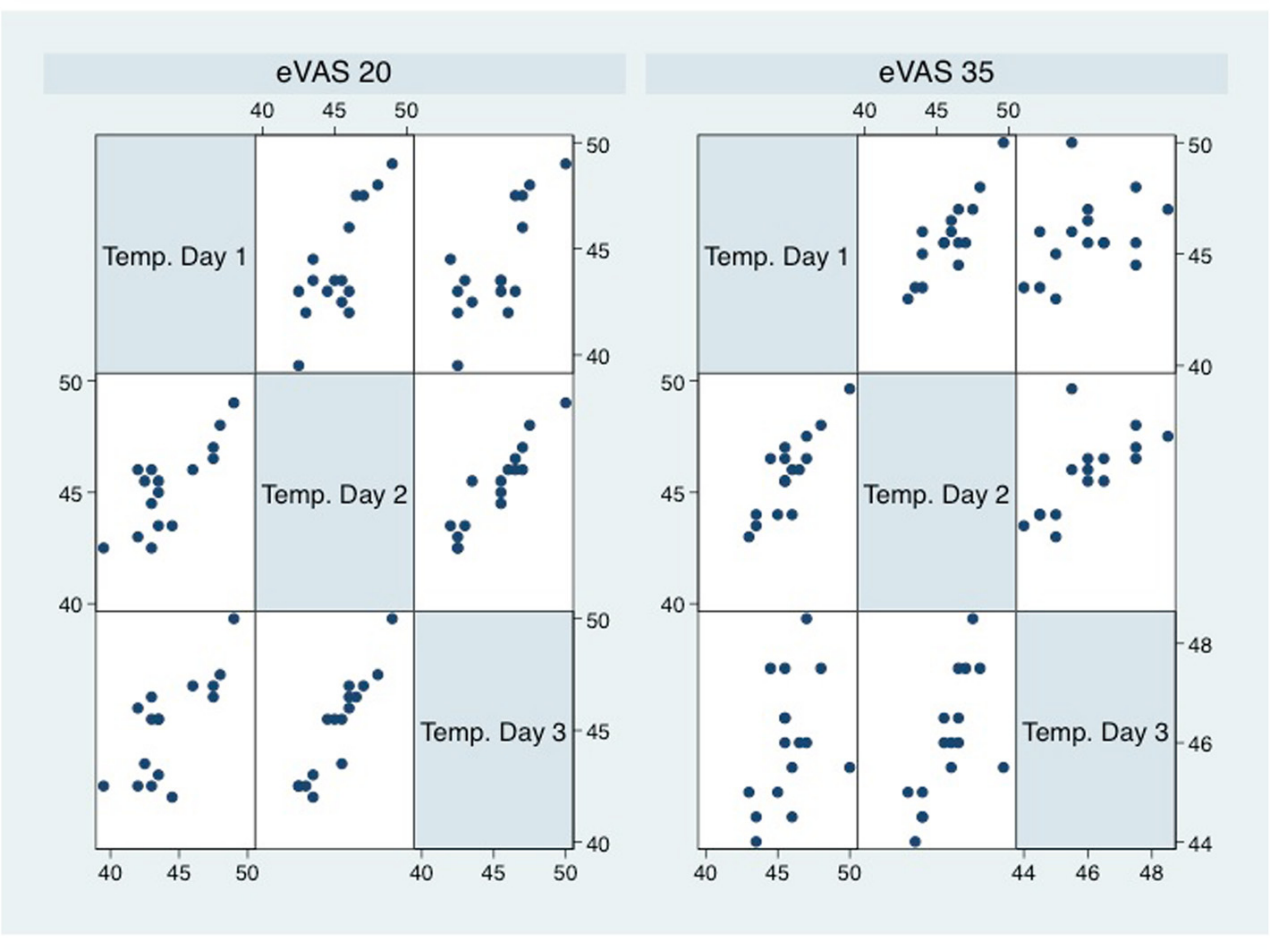
Average temperature correlations across the three days by pain set-point.

Intraclass correlation coefficients (ICC) for each of the variables of interest across the three days of testing are presented in Table 1. In summary, most variables derived from the output had reliability coefficients in the moderate to substantial range (r’s = 0.79 to 0.94) except for the negative AUC (r = 0.52), but only at the 20/100 pain rating set-point. Figure 3 shows all scatterplots across the three days of testing by each pain rating set-point.

**Table 1:** **Intraclass correlation coefficients along with the 95% confidence intervals for the measures derived from the REDSTIM output across 3 different days of testing**

	**eVAS = 20**	**Probability**	**eVAS = 35**	**Probability**
	**ICC (95%CI)**		**ICC (95%CI)**	
**Positive AUC**	0.79 (0.51-0.92)	p < 0.001	0.88 (0.71-0.96)	p < 0.001
**Negative AUC**	0.52 (0.00-0.60)	p = 0.041	0.80 (0.54-0.93)	p < 0.001
**Running Ave Temp: 60 sec**	0.90 (0.76-0.96)	p < 0.001	0.94 (0.85-0.98)	p < 0.001
**Running Ave Temp: 120 sec**	0.89 (0.74-0.96)	p < 0.001	0.92 (0.81-0.97)	p < 0.001

Positive AUC = Mean area under the curve of positive half-cycles.

Negative AUC = Mean area under the curve of negative half-cycles.

Running Ave Temp: 60 sec = Mean temperature across first 60 seconds of trial.

Running Ave Temp: 120 sec = Mean temperature across entire 120 seconds of trial.

A repeated measures ANOVA was also performed to compare the REDSTIM measures across days and eVAS pain ratings (20 and 35). The assumptions of normality and sphericity were met for all the variables examined. Results are summarized in Table 2. There were no statistically significant differences after correcting for multiple comparisons.

**Table 2:** **Means and standard deviations for the measures derived from the REDSTIM output across 3 different days of testing**

	**Day 1**	**Day 2**	**Day 3**
	**(Mean ± SD)**	**(Mean ± SD)**	**(Mean ± SD)**
**Positive AUC**			
**eVAS = 20**	79.6 ± 55.2	59.4 ± 35.5	74.1 ± 41.0
**eVAS = 35**	79.5 ± 51.1	65.6 ± 33.9	65.3 ± 39.0
**Negative AUC**			
**eVAS = 20**	86.8 ± 55.9	56.3 ± 36.2	62.9 ± 7.3
**eVAS = 35**	83.7 ± 52.5	61.8 ± 33.9	69.2 ± 41.5
**Running Ave Temp: 60 sec (°C)**			
**eVAS = 20**	44.5 ± 2.6	45.4 ± 2.1	45.7 ± 2.3
**eVAS = 35**	45.9 ± 1.8	46.1 ± 1.7	46.4 ± 1.8
**Running Ave Temp: 120 sec (°C)**			
**eVAS = 20**	44.9 ± 2.3	45.2 ± 1.8	45.5 ± 2.1
**eVAS = 35**	46.0 ± 1.7	45.9 ± 1.8	46.5 ± 1.9

Positive AUC = Mean area under the curve of positive half-cycles.

Negative AUC = Mean area under the curve of negative half-cycles.

Running Ave Temp: 60 sec = Mean temperature across first 60 seconds of trial.

Running Ave Temp: 120 sec = Mean temperature across entire 120 seconds of trial.

## Discussion

This is the first study designed to assess the test-retest reliability of REDSTIM tested at the thenar eminence of the palm over 120 seconds across three different days. The reliability coefficients were in the moderate to excellent range supporting its inter-session or test-retest repeatability. The only REDSTIM measure that had “fair” test-retest reliability occurred when using the 20/100 set-point. This may be due to floor effects (i.e., many participants getting down to 0/100 eVAS pain ratings). Given the importance of assessing pain sensitivity in a controlled and unbiased fashion for therapeutic efficacy trials, as well as the ability to measure effects of prolonged painful stimulation on the nociceptive system, REDSTIM has enormous potential for advancing the current state of clinical pain research and treatment. Self-report visual analog scales have previously shown excellent psychometric properties including reliability [8,9]. Quiton & Greenspan [4] also found that the variability of pain ratings in response to heat pain was stable within- and across- experimental sessions with the greatest variability at lower pain levels. However, previous studies have used conventional QST methodology without the ability to blind the participants, avoid direct subject-experimenter interactions and the inability to continuously adjust thermode temperature from the real-time eVAS ratings of the subjects like in this study’s REDSTIM methodology. Although it can be seen from Figure 1 the large between-subject variability in the temperature oscillations, these oscillations appear to be stable across three days.

The painful stimulation obtained with REDSTIM may better model clinical pain conditions where the pain experience remains at suprathreshold levels for extended periods of time. A stimulus that lasts for a few seconds may not reveal pain modulatory effects including sensitization and desensitization. Traditional methods of assessing sensitization have used short series of brief thermal stimuli of constant magnitude while measuring pain intensity changes [9,10]. These methods do not provide insight into slowly responding pain modulatory mechanisms because the stimulation series is short in order to keep the pain ratings from escalating to intolerable levels. The mechanisms underlying REDSTIM are not currently understood. Our previous work with REDSTIM also shows that even 2 minutes of very low pain may not be enough to address sensitization and desensitization [4]. Future studies elucidating the underlying mechanisms involved in the REDSTIM paradigm are needed including studies using higher temperature set-points and longer stimulation periods. However, the present study serves as a first step to address the reproducibility of this method in clinical and translational pain research.

There are several limitations in the present study that should be taken into consideration. First, the degree to which expectations impact the measures obtained from REDSTIM is not known. Continuous pain ratings require an increased focus on pain compared to conventional pain application, which may lead to a higher pain perception than unattented pain application. However, we provided standardized instructions to continuously rate sensation magnitude. Therefore, the subjects were not intentionally biased toward the ascending or descending series of stimulation. Future studies should be designed to examine whether sensitization and desensitization trends have common mechanisms with placebo and nocebo manipulations. Second, the current findings are only relevant to heat stimuli and other stimuli modalities may yield different findings. Third, the current study tested only mild to moderate pain intensity set-points, thus the stability of patterns at moderate to severe pain intensity set-points is not known. Finally, the present study was carried out in healthy young participants. It will be important to examine the stability of the REDSTIM paradigm in older individuals and those with clinical pain conditions where the chronic pain may vary considerably over time.

## Conclusions

Despite its limitations, our study supports the repeatability and stability of REDSTIM measures, which has important implications for clinical pain research studies. The REDSTIM paradigm may provide new insights into the mechanisms underlying chronic pain, which often fluctuates at various intensity levels. Unlike conventional methods, REDSTIM blinds the subject regarding the pain intensity set-point, supporting the future use of REDSTIM for assessing intervention efficacy. By maintaining the pain intensity at a constant level with the REDSTIM paradigm, a potential decrease in pain sensitivity can be detected by an increase in thermode temperature (unknown to the subject) and not by the pain ratings alone. Finally, the prolonged stimulation in REDSTIM may provide a novel insight into human pain mechanisms while overcoming common shortcomings of conventional QST methods.

## Methods

### Participants

Recruitment and study procedures were approved by the University of Florida Institutional Review Board. Written informed consent was obtained from all participants. The volunteers were required to have (1) no significant spontaneous pain anywhere in the body; (2) no ongoing pharmacotherapy with narcotics or antidepressants; (3) no disease that might significantly affect pain perception or unduly increase risk of injury (e.g., neurological disorders, serious psychiatric disorders, diabetes, hypertension, serious cardiovascular disorders, and chronic pain). Twelve of the sixteen participants were included in a previous study [1].

### Pain measurement

Experimental pain was measured with an electronic version of a visual analog scale [11]. The electronic visual analog scale (eVAS) consisted of a low-friction sliding potentiometer of 100 mm travel. The left endpoint of the scale was identified as “no pain”, while the right endpoint was defined as “intolerable pain”. There were no divisions between these two anchors. The position of the slider was electronically converted into a pain rating between 0 and 100%. The slider automatically returned to the left (“no pain”) position when so required by the protocol. The eVAS was mounted into the surface of a small inclined desk positioned to facilitate precise operation with minimal fatigue. The custom-built testing system (Neuroanalytics Corporation, Gainesville, FL) integrated all inputs (temperature process value, eVAS signal) and outputs (stimulus temperature control, stimulus timing) and allowed automated execution of test protocols with preprogrammed parameters, including limits for temperature.

### Response-Dependent Stimulation (REDSTIM) method

Thermal stimuli were administered with a flat copper contact thermode of 23x23mm in size. The thermode was electronically held at the desired temperature by a Peltier thermoelectric device. It was brought into light skin contact of reproducible force by solenoid activation, which was preprogrammed in the stimulator control software, allowing fully automated data collection. A pain intensity set-point was defined, and an algorithm in the stimulator control software calculated the deviation of the patient’s actual pain rating from the set-point as well as the derivative of this error. These data were the basis for automatic adjustments of the stimulus temperature to maintain an average pain rating that equaled the set-point.

In summary, REDSTIM is composed of the induction and the maintenance phase. During the induction phase, the thermode temperature was increased from non-painful levels (35°C) with temperature steps decreasing in size as pain ratings approach the set-point. During the induction phase, the temperature could increase or stay the same, but never decrease. Once pain intensity reached the set-point the maintenance phase began. When pain intensity exceeded the set-point the temperature was stepped down. Conversely, when the pain intensity was below the set-point, the stimulus temperature was stepped up. A temperature limit is set to prevent thermal injury of subjects that are pain insensitive. More detailed information on the system, software and paradigm has been previously reported elsewhere [1,2]. For the present study, continuous heat application was used to examine the psychometric properties of the oscillations occurring during the REDSTIM methodology.

### Testing protocol

Thermal stimulation was conducted during three separate and identical daily sessions each separated by two-days apart. During each session REDSTIM experiments were conducted on the thenar eminence of the hand. Two set-points were tested on each subject in every session: 20/100 and 35/100 pain ratings. REDSTIM oscillations around the set-point were sampled every 1.5 seconds for a period of 120 seconds during all sessions and at both 20/100 and 35/100 set-point parameters. We used mild to moderate pain rating set-points to avoid reaching intolerable levels of pain in the most sensitive subjects. All test conditions were the same for the three experimental sessions and subjects were not informed of the pain rating set-points used.

Pain ratings above the set-point progressed in positive half-cycles that began within ascending temperature progressions, progressed to a high peak pain rating and then returned to the set-point. Similarly, pain ratings below the set-point in negative half-cycles began within descending temperature progressions progressed to a low peak pain rating and then returned to the set-point. For each half-cycle (positive and negative), deviations from the set-point were summed over time and the average area under the curve (AUC) was calculated for each subject for each day of testing. In addition, the software program automatically calculates the average temperature as the cycle progresses. The output provides a running average temperature needed to maintain the set-point at 60 seconds (i.e., for the first 60 seconds of the trial) and at 120 seconds of the cycle (i.e., average of the entire trial). Test-retest reliability was calculated for each of these variables.

### Data analysis

Measures must have at least “Fair” test-retest reliability (ICC > 0.41) in order to be acceptable for its use, but ideally coefficients should fall within the “Moderate” (ICC > 0.61) to “Substantial” (ICC > 0.81) range [6,12]. Based on this premise, we estimated that a sample size of 16 subjects was required to measure an intra-class correlation coefficient (ICC) of at least 0.61 across three days, with a power of at least 0.80 at an alpha level of 0.05. We also assessed differences between days using a repeated measures ANOVA with “Days” and “eVAS set-point” (20 or 35) as within-subjects variables. First, data were examined for distribution, presence of extreme outliers and the Mauchly’s test of Sphericity was performed. If the sphericity assumption was violated, then Greenhouse-Geisser degrees of freedom corrections were applied. In addition, Bonferroni was used to adjust for multiple comparisons. Statistical analyses were conducted with IBM SPSS 22 for Windows. For all tests, 2-sided p values less than 0.05 were considered statistically significant.
